# Medical resource utilization among patients with ventilator-associated pneumonia: pooled analysis of randomized studies of doripenem versus comparators

**DOI:** 10.1186/cc9012

**Published:** 2010-05-10

**Authors:** Marin H Kollef, Dilip Nathwani, Sanjay Merchant, Christopher Gast, Alvaro Quintana, Nzeera Ketter

**Affiliations:** 1 Department of Medicine, Washington University School of Medicine, 660 South Euclid Avenue, St. Louis, Missouri 63110, USA; 2 Infection Unit, East Block, Level 4, Ninewells Hospital and Medical School, Dundee DD1 9SY, UK; 3 Worldwide Health Economics & Pricing, Johnson and Johnson Pharmaceutical Services, LLC, 700 Route 202 Raritan, New Jersey 08869-0602, USA; 4 Axio Research Corporation LLC, 2601 4th Avenue, Suite 200, Seattle, WA 98121, USA; 5 Current address: Private consultant, Seattle, WA, USA; 6 Anti-Infectives, Johnson and Johnson Pharmaceutical Services, LLC, 700 Route 202, Raritan, New Jersey 08869-0602, USA; 7 Anti-Infectives, Johnson and Johnson Research and Development, 6500 Paseo Padre Parkway, MS: B-1, Fremont, California 94555, USA

## Abstract

**Introduction:**

Ventilator-associated pneumonia (VAP) is associated with increased medical resource utilization, but few randomized studies have been conducted to evaluate the effect of initial antibiotic therapy. To assess medical resource utilization in patients with VAP, we conducted a pooled analysis of two prospective, randomized, open-label, multicenter, phase III studies, which also showed that doripenem was clinically noninferior to comparators.

**Methods:**

We assessed durations of mechanical ventilation, intensive care unit (ICU) stay, and hospitalization in patients with VAP who received at least 1 dose of doripenem or a comparator in the phase III studies. Comparators were piperacillin/tazobactam (study 1) and imipenem (study 2). We analyzed between-group differences in medical resource utilization endpoints by comparison of Kaplan-Meier curves with generalized Wilcoxon test and in microbiologic eradication rates by two-sided Fisher's exact test.

**Results:**

625 patients with VAP were evaluated and received at least 1 dose of doripenem (n = 312) or a comparator (n = 313). Median durations of mechanical ventilation (7 versus 10 days; *P *= 0.008) and hospitalization (22 versus 26 days; *P *= 0.010) were shorter for doripenem than comparators; corresponding ICU stays were 12 and 13 days (*P *= 0.065). All-cause, overall mortality rates were similar (51/312 [16%] versus 47/313 [15%]; *P *= 0.648). MIC_90 _values against *Pseudomonas aeruginosa *for doripenem versus imipenem were 4 versus 16 μg/mL in study 2. *P. aeruginosa *was eradicated from 16/24 (67%) doripenem recipients and 10/24 (42%) comparator recipients (*P *= 0.147). In patients with *P. aeruginosa *at baseline, median durations of mechanical ventilation (7 versus 13 days; *P *= 0.031) and ICU stay (13 versus 21 days; *P *= 0.027) were shorter for doripenem; corresponding hospital stays were 24 and 35 days (*P *= 0.129).

**Conclusions:**

Doripenem was associated with lower medical resource utilization than comparators. Differences in antipseudomonal activity may have contributed to these findings.

**Trial registration:**

ClinicalTrials.gov number NCT00211003 (study 1) and NCT00211016 (study 2).

## Introduction

Ventilator-associated pneumonia (VAP) imposes a burden on medical resources, with attributable hospital costs ranging from $10,000 to $12,000 per episode [1-3]. Length of stay (LOS) is an important component of hospital costs. VAP adds five days to the duration of mechanical ventilation [1], four to six days to LOS in the ICU [2,4], and seven days to overall duration of hospitalization [1]. LOS is even longer in patients with VAP due to microorganisms that are more virulent, such as *Pseudomonas aeruginosa *[4-6], or that are resistant to imipenem [7] or multiple drugs [8,9].

Appropriate initial antibiotic therapy is vital because delayed treatment is associated with increased risk of mortality [4,10-15]. The choice of therapy depends on the presence of risk factors for multidrug-resistant pathogens and time of VAP onset. Patients with risk factors or late-onset VAP are at increased risk of infection due to *P. aeruginosa *and are therefore candidates for an antipseudomonal carbapenem [16].

Doripenem is a broad-spectrum carbapenem with activity against *P. aeruginosa *[17]. In phase III studies, doripenem was clinically noninferior compared with piperacillin/tazobactam in patients with nosocomial pneumonia (study 1) [18] and with imipenem in patients with VAP (study 2) [19]. Additionally, doripenem was associated with shorter durations of mechanical ventilation and hospitalization than was imipenem in study 2; between-group differences in ICU LOS were not significant [20].

Few randomized studies have been conducted to prospectively evaluate the effect of initial antibiotic therapy on medical resource utilization, such as duration of mechanical ventilation, ICU LOS, and hospital LOS. To compare medical resource utilization for doripenem with that for comparators, we conducted a pooled analysis of studies 1 [18] and 2 [19]. Considering the added burden of *P. aeruginosa *on resource utilization [4-6], we also evaluated between-group differences in microbiologic outcome and medical resource utilization in patients with *P. aeruginosa *at baseline.

## Materials and methods

Data for this pooled analysis were obtained from two prospective, randomized, open-label, multicenter, phase III studies, which were conducted between June 2004 and October 2006 to evaluate whether doripenem was noninferior to comparator drugs. Study 1 was conducted at 24 centers in North America, 18 in South America, and 26 in Europe; study 2 was conducted at 37 centers in North America, 33 in Western Europe, 11 in Australia, and 3 in other parts of the world. Study designs have been previously reported [18-20] and were similar, unless otherwise indicated. Adults were eligible for study 1 if they had clinical and radiologic criteria for nosocomial pneumonia and early-onset VAP, defined as less than five days of mechanical ventilation and a Luna Clinical Pulmonary Infection Score of 5 or more [21]; the pooled analysis included only the subset with VAP. Adults were eligible for study 2 if they had VAP as previously defined. Study protocols and informed consent forms were reviewed and approved by an institutional review board or ethics committee at each study center. All patients or their legally appointed representatives provided written informed consent.

Eligible patients were stratified by non-VAP and early VAP in study 1, early and late VAP in study 2 (onset <5 versus ≥5 days), geographic region, and Acute Physiology and Chronic Health Evaluation (APACHE) II scores (≤15 versus >15). After stratification, patients were randomly assigned to receive doripenem 500 mg every eight hours or a comparator, each intravenously. Doripenem was infused over one hour in study 1 and over four hours in study 2. In study 1, the comparator was piperacillin/tazobactam 4.5 g every six hours infused over 30 minutes; patients who were clinically improved after at least 72 hours of intravenous therapy could be switched to oral levofloxacin 750 mg daily. In study 2, the comparator was imipenem 500 mg every six hours infused over 30 minutes or 1000 mg every eight hours infused over one hour. Both studies allowed adjunctive therapy with vancomycin for suspected methicillin-resistant *Staphylococcus aureus *(MRSA) or an aminoglycoside for suspected *P. aeruginosa*. All antibiotic dosages were adjusted for renal function; antibiotic concentrations were not collected.

Endpoints were prospectively defined in the phase III study protocols. Medical resource utilization, which was retrospectively analyzed from prospectively collected data in the pooled analysis, included durations of mechanical ventilation, ICU stay, and hospitalization. Duration of mechanical ventilation was defined as stop date - maximum (start date or randomization date) + 1. If the stop date was not available, the minimum of the following was used for censoring: death, ICU discharge, hospital discharge, or late follow-up, which occurred 28 to 35 days after the end of intravenous therapy. Duration of ICU stay was defined as ICU discharge date - maximum (ICU admit date or randomization date) + 1. Nine patients were excluded from ICU analysis because they had valid ICU admittance dates but no valid ICU discharge dates and had hospital discharge dates (doripenem, 3; comparator, 6). Duration of hospitalization was defined as (discharge date or death date) - randomization date + 1. If discharge date was not available, patients were censored at late follow up. In addition, all-cause overall mortality and, in patients with *P. aeruginosa *at baseline, minimal inhibitory concentrations (MIC), eradication rate, and resource utilization were evaluated.

### Statistical analyses

Medical resource utilization endpoints were analyzed in all patients who received at least one dose of study drug, defined as the intent-to-treat (ITT) population, and who met the clinical definition of VAP (clinical modified ITT) [19]. Microbiologic endpoints were analyzed in patients from whom *P. aeruginosa *alone or with other microorganisms had been isolated at baseline from the lower respiratory tract.

Between-group differences in medical resource utilization endpoints were analyzed by comparison of Kaplan-Meier curves with Gehan's generalization of the Wilcoxon test [22], whose weighting scheme reduces the relative contribution of patients with prolonged LOS due to other (non-VAP) conditions by placing more weight on early versus later discharges. Between-group differences in microbiologic eradication rates were analyzed by two-sided Fisher's exact test and in LOS by generalized Wilcoxon test.

Effect of covariates on outcome variables, as well as treatment effect after controlling for important covariates, was examined with Cox proportional hazards regression model. Variables were selected by the best-subsets model-selection method, where the smallest model with statistically significant variables was included; the *P *value for inclusion in the model was less than 0.05. In addition, baseline microbiology of *P. aeruginosa *or MRSA was included as a clinically important variable. Assumption of proportional hazards was tested in each case by examining the effect-by-time interaction effect jointly for all covariates, and individually for each covariate entered into the model. The proportional hazards assumption was validated for each model.

Statistical Analysis Software version 9.1.3 (SAS Institute, Inc., Cary, NC, USA) was used for statistical analyses. *P *values less than 0.05 were considered statistically significant.

## Results

**Figure 1:**
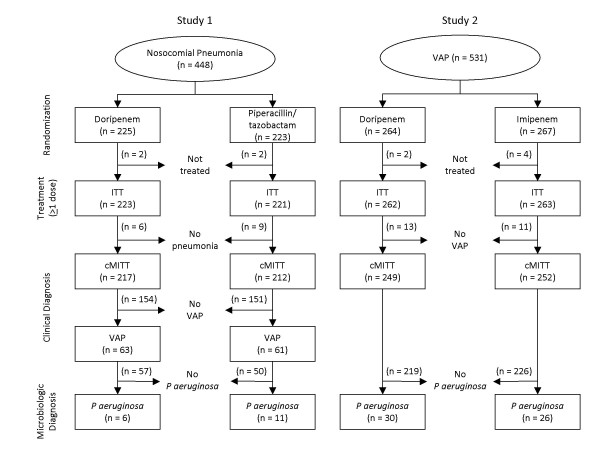
**Patient disposition**. cMITT, clinically modified ITT (population); ITT, intent-to-treat (population); VAP, ventilator-associated pneumonia.

Of 979 patients randomized to receive doripenem (n = 489) or a comparator (n = 490) in studies 1 and 2 (Figure 1), 354 were excluded from the pooled analysis because they did not receive the study drug (n = 10) or did not meet clinical diagnostic criteria for VAP (n = 344). Most of these exclusions occurred in patients enrolled in study 1 who had nosocomial pneumonia but not VAP. In the remaining 625 patients, none of the tests for between-group differences in demographics, clinical characteristics, and adjunctive medication use yielded statistically significant results (Table 1). All remaining patients were included in the pooled analysis except four who received comparators and did not have valid medical resource utilization data.

**Table 1:** Demographics, clinical characteristics, and drug use for study patients^a^

Characteristic	Number of patients (%), unless otherwise stated					
	
	Study 1		Study 2		Pooled^b^	
			
	Doripenem (n = 63)	Pip/Tazo (n = 61)	Doripenem (n = 249)	Imipenem (n = 252)	Doripenem (n = 312)	Comparator (n = 313)
Mean age, years (SD)	50.8 (20.1)	54.0 (20.4)	51.4 (19.8)	51.7 (18.7)	51.3 (19.8)	52.2 (19.0)
Men	42 (66.7)	46 (75.4)	195 (78.3)	192 (76.2)	237 (76.0)	238 (76.0)
APACHE II ≤15	35 (55.6)	32 (52.5)	117 (47.0)	120 (47.6)	152 (48.7)	152 (48.6)
Race						
White	39 (61.9)	40 (65.6)	217 (87.1)	209 (82.9)	256 (82.1)	249 (79.6)
Black	7 (11.1)	3 (4.9)	22 (8.8)	28 (11.1)	29 (9.3)	31 (9.9)
Hispanic	16 (25.4)	17 (27.9)	9 (3.6)	10 (4.0)	25 (8.0)	27 (8.6)
Other	1 (1.6)	1 (1.6)	1 (0.4)	5 (2.0)	2 (0.6)	6 (1.9)
VAP onset						
Early (<5 days)	63 (100.0)	61 (100.0)	98 (39.4)	97 (38.5)	161 (51.6)	158 (50.5)
Late (≥5 days)	0 (0.0)	0 (0.0)	151 (60.6)	155 (61.5)	151 (48.4)	155 (49.5)
Adjunctive drug usage						
Amikacin	52 (82.5)	51 (83.6)	28 (11.2)	40 (15.9)	80 (25.6)	91 (29.1)
Vancomycin	18 (28.6)	13 (21.3)	70 (28.1)	74 (29.4)	88 (28.2)	87 (27.8)
Oral levofloxacin	9 (14.3)	7 (11.5)	Not applicable		9 (2.9)	7 (2.2)
Baseline microbiology^c^						
*Pseudomonas aeruginosa*	6 (9.5)	11 (18.0)	30 (12.0)	26 (10.3)	36 (11.5)	37 (11.8)
MRSA	6 (9.5)	3 (4.9)	14 (5.6)	16 (6.3)	20 (6.4)	19 (6.1)
Surgery	45 (71.4)	41 (67.2)	193 (77.5)	194 (77.0)	238 (76.3)	235 (75.1)

^a ^Patients who received one dose or more of study drug and met clinical diagnostic criteria for VAP.

^b ^*P *> 0.05, doripenem versus comparator.

^c ^Percentages were based on all patients, including some patients with unknown baseline microbiology.

APACHE, Acute Physiology and Chronic Health Evaluation; MRSA, methicillin-resistant *Staphylococcus aureus*; Pip/Tazo, piperacillin/tazobactam; SD, standard deviation; VAP, ventilator associated pneumonia.

**Figure 2:**
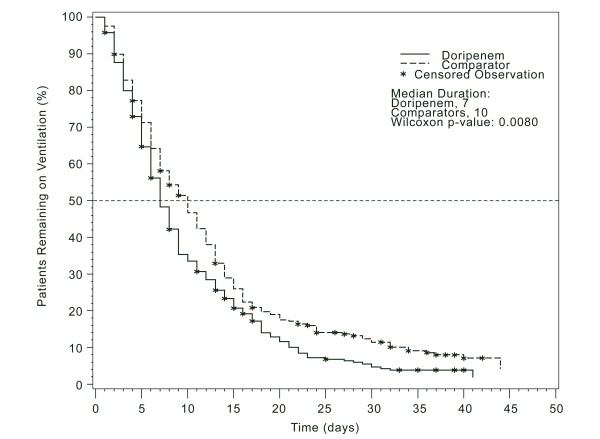
**Kaplan-Meier curve of duration of mechanical ventilation**. Asterisks represent censored observations.

Durations of mechanical ventilation and hospitalization were shorter among patients treated with doripenem than among those treated with a comparator; between-group differences in ICU stay were not statistically significant (Table 2). For example, median duration of mechanical ventilation was 7 days for doripenem and 10 days for comparators; the *P *value for the Kaplan-Meier curve comparison was 0.008 (Figure 2). Similar trends were seen for the subset of patients who either survived or died seven or more days after stopping mechanical ventilation. All-cause, overall mortality occurred in 51 (16%) of 312 patients in the doripenem group and in 47 (15%) of 313 patients in the comparator group (*P *= 0.648).

**Table 2:** Medical resource utilization in VAP patients who received at least one dose of study drug

Type of resource	Median duration in days (95% CI), unless otherwise stated		*P *Value
		
	Doripenem	Comparator	
All patients	(n = 312)^a^	(n = 309)^a^	
Mechanical ventilation	7 (7-8)	10 (8-11)	0.008
ICU	12 (11-13)	13 (12-16)	0.065
Hospital	22 (20-25)	26 (23-29)	0.010
Patients who survived or died ≥7 days after stopping mechanical ventilation	(n = 282)^a^	(n = 270)^a^	
Mechanical ventilation	7 (7-8)	9 (7-11)	0.053
ICU	13 (11-14)	14 (12-16)	0.162
Hospital	23 (21-27)	28 (26-32)	0.004
Mortality, n/N (%)	51/312 (16.3)	47/313 (15.0)	0.648

^a ^Number of patients assessed for duration of hospitalization. Some patients had missing data for durations of mechanical ventilation and ICU stay.

CI, confidence interval; VAP, ventilator-associated pneumonia.

In the Cox proportional hazards model, patients in the doripenem group were 1.3 times more likely to be weaned from mechanical ventilation (*P *= 0.005) or discharged from the hospital (*P *= 0.004) than those in the comparator group (Table 3). Hazard ratios for treatment with doripenem versus comparators corresponding to risk of stopping mechanical ventilation (*P *= 0.006) and hospital discharge (*P *= 0.004) remained significantly above one after adjusting for other significant covariates, such as presence of *P. aeruginosa*. The hazard ratio corresponding to risk of ICU discharge was not significantly different from one before (*P *= 0.079) or after (*P *= 0.122) adjusting for significant covariates.

**Table 3:** Cox proportional hazards regression for medical resource utilization

Parameter	Hazard ratio^a^	95% Confidence interval	*P *value
Duration of mechanical ventilation			
Unadjusted results			
Treatment (doripenem)	1.29	1.08-1.53	0.005
Adjusted results			
Treatment (doripenem)	1.28	1.07-1.52	0.006
Baseline MRSA (presence)	0.72	0.50-1.04	0.079
Baseline *Pseudomonas aeruginosa *(presence)	0.79	0.60-1.04	0.087
Baseline APACHE II score^b ^(≤20 versus >20)	0.77	0.61-0.95	0.017
ICU LOS			
Unadjusted results			
Treatment (doripenem)	1.16	0.98-1.37	0.079
Adjusted results			
Treatment (doripenem)	1.14	0.97-1.35	0.122
Baseline *P. aeruginosa *(presence)	0.73	0.56-0.95	0.018
Region (North America)	1.22	1.04-1.45	0.018
Baseline APACHE II score	0.97	0.96-0.99	0.003
Total hospital LOS			
Unadjusted results			
Treatment (doripenem)	1.32	1.09-1.58	0.004
Adjusted results			
Treatment (doripenem)	1.31	1.09-1.58	0.004
VAP onset (early)	1.30	1.08-1.57	0.005
Baseline *P. aeruginosa *(presence)	0.83	0.62-1.11	0.212
Region (North America)	1.51	1.25-1.82	<0.001

^a ^A hazard ratio significantly greater than 1 indicated that discharge was more likely to occur.

^b ^APACHE II score was dichotomous (not continuous as in previous models) to satisfy the proportional hazards assumption.

APACHE II, Acute Physiology and Chronic Health Evaluation II; LOS, length of stay; MRSA, methicillin-resistant *Staphylococcus aureus*; VAP, ventilator-associated pneumonia.

**Figure 3:**
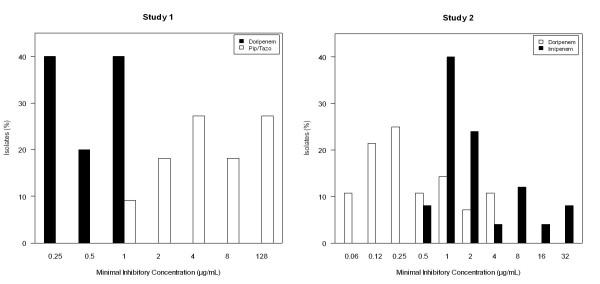
**Distribution of minimal inhibitory concentrations for *Pseudomonas aeruginosa***. The number of isolates was 5 for doripenem and 11 for piperacillin/tazobactam in study 1, and 28 for doripenem and 25 for imipenem in study 2.

In the subset of patients with *P. aeruginosa *at baseline, this pathogen was eradicated or presumed eradicated from 16 (67%) of 24 patients in the doripenem group and from 10 (42%) of 24 patients in the comparator group (*P *= 0.147; Table 4). In study 1, MIC_50 _values were 0.5 μg/mL for doripenem and 4.0 μg/mL for piperacillin/tazobactam, and corresponding MIC_90 _values were 1 and 128 μg/mL (Figure 3). In study 2, MIC_50 _values were 0.25 μg/mL for doripenem and 2.0 μg/mL for imipenem, and corresponding MIC_90 _values were 4 and 16 μg/mL. Median durations of mechanical ventilation (7 versus 13 days; generalized Wilcoxon *P *= 0.031) and ICU stay (13 versus 21 days; *P *= 0.027) were shorter for doripenem; between-group differences in hospital stay were not statistically significant.

**Table 4:** Microbiologic outcome and resource utilization in patients with ventilator-associated pneumonia due to *Pseudomonas aeruginosa*

Outcome	Doripenem	Comparator	*P *value
Eradication rate, n/N (%)^a^	16/24 (66.7)	10/24 (41.7)	0.147^b^
Median duration, days (95% CI)	(n = 36)	(n = 37)	
Mechanical ventilation	7 (5-9)	13 (8-16)	0.031^c^
ICU	13 (9-19)	21 (14-30)	0.027^c^
Hospital	24 (20-32)	35 (28-N/A)	0.129^c^

^a ^Eradication rate defined as microbiologic eradication or presumed eradication. Twenty-five patients excluded from evaluation of eradication rates for doripenem (n = 12) and comparator (n = 13) because of lack of data.

^b ^Two-sided Fisher's exact test.

^c ^Generalized Wilcoxon test.

CI, confidence interval; N/A, not available.

## Discussion

The results of this pooled analysis of two phase III studies indicated that initial use of doripenem in patients with VAP was associated with shorter durations of mechanical ventilation and hospitalization than was use of comparator antibiotics. Between-group differences in hospital LOS remained significant in the subset of patients who survived or died seven or more days after stopping mechanical ventilation, suggesting that the difference was not due to an imbalance in mortality. Treatment group was associated with durations of mechanical ventilation and hospitalization after adjusting for other significant covariates in the regression analysis. Median duration of mechanical ventilation in the comparator group (10 days) was generally consistent with that in previous cohort studies of patients with VAP (10 to 14 days) [1,23]. Similarly, duration of hospitalization (26 days) was within the range in previous studies (15 to 38 days) [1,3,23].

To further elucidate our findings, we evaluated patients with *P. aeruginosa *at baseline. Microbiologic eradication rates were 67% for doripenem and 42% for comparators; however, the between-group difference was not statistically significant, presumably because of the limited number of patients in this subset. We also found that doripenem had lower MIC values than did imipenem. Comparison of MIC values among different antibiotic classes are not an indication of clinical efficacy; however, free time above MIC in serum (*f*T>MIC) can be used as a surrogate for comparison. This pharmacokinetic/pharmacodynamic index correlates with clinical efficacy and bactericidal activity and is used to determine antibiotic dosage regimens [24,25]. Pharmacodynamic modeling reveals that the doripenem dosages would have a 99 to 100% probability of achieving a target *f*T>MIC for the MIC_90 _values in study 1 (1 μg/mL) and study 2 (4 μg/mL) [26]. In contrast, the piperacillin/tazobactam dosage would have a very low probability of achieving a target *f*T>MIC for the MIC_90 _(128 μg/mL) in study 1 [27].

Finally, we found significant between-group differences in medical resource utilization favoring doripenem in patients with *P. aeruginosa *at baseline for duration of mechanical ventilation and also for ICU LOS, whereas that for hospital LOS was not significant. As expected, each measure of medical resource utilization was greater for patients in the comparator group with *P. aeruginosa *at baseline than for those without *P. aeruginosa*. For example, median duration of mechanical ventilation was 10 days for all patients with VAP and 13 days for the subset with *P. aeruginosa *at baseline. Vidaur and colleagues [5] reported that, when patients were treated with inappropriate antibiotics, clinical resolution occurred more slowly if VAP was due to *P. aeruginosa *than due to other pathogens. Interestingly, each measure of medical resource utilization was nearly identical for patients in the doripenem group, regardless of whether *P. aeruginosa *was present. Therefore, these findings suggest that doripenem may have prevented the increased medical resource utilization associated with *P. aeruginosa *at baseline reported in other studies [4-6] because of activity against key pathogens. Consideration of the influence of appropriateness of empiric therapy against subsequently identified pathogens was beyond the scope of this economic analysis and merits further evaluation.

The lack of statistically significant between-group difference in one of three measures of medical resource utilization is puzzling, given the relation between the outcome measures. In the subset with *P. aeruginosa *at baseline, the absolute difference in hospital LOS was nine days. The absolute difference in ICU stay was only one day in all patients and is more difficult to explain. During acute critical illness requiring ICU stay of more than 48 hours, health-related quality of life (HRQOL) declines rapidly. After patients are discharged from the ICU, HRQOL begins to recover and reaches near pre-ICU admission levels by the time of hospital discharge [28]. Therefore, an intervention with a more rapid effect on VAP may allow patients to recover more quickly on the ward and to be discharged from the hospital more quickly. This hypothesis requires further evaluation with repeated assessment of HRQOL throughout hospitalization and after discharge.

Our findings address a gap in the literature regarding the effect of initial antibiotic therapy on medical resource utilization in patients with VAP. The larger, combined sample provided a sufficient number of patients with *P. aeruginosa *at baseline to show a statistically significant difference between those who received doripenem and those who received a comparator, which was not detected in a previous study containing only a portion of the combined sample [20]. These between-group differences may have clinical implications. Specifically, shortening LOS may decrease the cost of hospitalization as demonstrated in a recent economic analysis of studies 1 and 2 [29]. Using discrete event simulation, Kongnakorn and colleagues [29] estimated that use of doripenem instead of imipenem for treatment of VAP would yield a per-patient saving of approximately $12,260 primarily due to reduced durations of mechanical ventilation, ICU stay, and hospitalization. In addition, shortening LOS may decrease the risk of nosocomial complications, such as exposure to resistant microorganisms and recurrent VAP. Carmeli and colleagues [30] were among the first to report the economic burden of antibiotic resistance in *P. aeruginosa*. Emergence of resistance during hospitalization was associated with longer hospital LOS and increased risk of secondary bacteremia in their cohort study [30].

Our study had several limitations. First, this was a *post hoc *analysis, although data were collected prospectively and analysis of medical resource utilization was intended for the individual studies. Second, both study designs were open label, which may have affected medical resource utilization; however, bias should be minimal because the decision of whether to discharge patients or discontinue mechanical ventilation was likely to be based on overall health and predefined institutional procedures rather than treatment assignment, and the number of investigators was large.

Third, the studies were not identical, but they were similar except for the following. Study 1 excluded patients with late-onset VAP; study 2 included patients with early- and late-onset VAP. Study 1 (n = 123) had fewer patients than study 2 (n = 501). Doripenem was infused over one hour in study 1 and four hours in study 2. Comparators were piperacillin/tazobactam in study 1 and imipenem in study 2. Fourth, excluding patients without VAP from study 1 may have partially offset the benefits of randomization in that study; however, all patients in the clinically modified ITT population were included from study 2, which was larger than study 1.

Fifth, not all LOS was attributable to VAP. Patients could have been admitted for other reasons and subsequently acquired VAP. Furthermore, patients with VAP are critically ill and have other comorbidities that contribute to LOS. Additionally, we did not capture data needed to eliminate the possibility that social circumstances or other factors led to earlier discharge of patients who received doripenem, but randomization should have offset any between-group differences. Also, we used a statistical test that places more weight on early discharges than on late discharges and did not consider ICU readmissions. Sixth, the analyses do not prove a cause-and-effect relation between doripenem and shorter LOS, but statistical models were used to address covariates likely to influence treatment and outcome.

## Conclusions

This pooled analysis of two prospectively randomized phase III studies suggests that doripenem use for the treatment of VAP was associated with shorter durations of mechanical ventilation and hospitalization. Differences in antipseudomonal activity may have contributed to these findings. Our economic findings, combined with previous clinical findings, suggest that doripenem may be considered an alternative for empirical treatment of VAP, especially when *P. aeruginosa *is suspected or prevalent. More studies are needed to confirm these preliminary findings and to define the clinical and economic value of doripenem in patients with VAP.

## Key messages

• Initial doripenem use in patients with VAP was associated with statistically significant shorter durations of mechanical ventilation (median, 7 versus 10 days) and hospitalization (median, 22 versus 26 days) than was use of comparator antibiotics (piperacillin/tazobactam or imipenem) in a pooled analysis of two phase III studies.

• Initial doripenem use was also associated with statistically significant shorter durations of mechanical ventilation (median, 7 versus 13 days) and ICU stay (median, 13 versus 21 days) in the subset with *P. aeruginosa *at baseline.

• The economic findings from this paper, combined with previous clinical findings, suggest that doripenem may be considered an alternative for empirical treatment of VAP, especially when *P. aeruginosa *is suspected or prevalent.

## Abbreviations

APACHE: Acute Physiology and Chronic Health Evaluation; *f*T>MIC: free time above MIC in serum; HRQOL: health-related quality of life; ITT: intent to treat; LOS: length of stay; MIC: minimal inhibitory concentration; MRSA: methicillin-resistant *Staphylococcus aureus*; VAP: ventilator-associated pneumonia.

## Competing interests

This study, including statistical analysis and manuscript preparation, was supported by a grant from Johnson and Johnson Pharmaceutical Services, LLC. MK reported receiving a consulting fee from Kimberly Clark and lecture fees and grant support from Bard, Elan, Merck, Ortho-McNeil (lecture fees only), and Pfizer. DN reported receiving speaking fees and honoraria for meetings and advisory boards supported by Astellas, Wyeth, Bayer, Pfizer, and Johnson and Johnson (J&J). MK and DN have not received any fees for involvement in this study or manuscript. SM, AQ, and NK are employed by Johnson and Johnson. CG is employed by Axio, which received payment from Johnson and Johnson for its services.

## Authors' contributions

MK had full access to all of the data in the study and takes responsibility for the integrity of the data and the accuracy of the data analysis. MK contributed to study concept and design, to analysis and interpretation of data and to drafting of the manuscript and critical revision for important intellectual content. DN contributed to study concept and design, to analysis and interpretation of data and to drafting of the manuscript and critical revision for important intellectual content. SM contributed to study concept and design, to analysis and interpretation of data and to drafting of the manuscript and critical revision for important intellectual content. CG contributed to study concept and design, to analysis and interpretation of data, to drafting of the manuscript and critical revision for important intellectual content and to statistical analysis. AQ contributed to study concept and design, to analysis and interpretation of data and to drafting of the manuscript and critical revision for important intellectual content. NK contributed to study concept and design, to analysis and interpretation of data and to drafting of the manuscript and critical revision for important intellectual content.
